# Optical Quality Degradation Following Nd:YAG Laser-Induced Intraocular Lens Pitting: A Multimodal Experimental Study

**DOI:** 10.3390/vision10030054

**Published:** 2026-08-18

**Authors:** Laura De Luca, Feliciana Menna, Stefano Lupo, Elisa Ruello, Barbara Testagrossa, Giuseppe Acri, Matteo Mario Carlà, Antonio Baldascino, Enzo Maria Vingolo, Pasquale Aragona, Alessandro Meduri

**Affiliations:** 1 Ophthalmology Clinic, Department of Biomedical Sciences, University of Messina, 98122 Messina, Italy; 2 Department of Medical-Surgical Sciences and Biotechnologies, U.O.C. Ophthalmology, Sapienza University of Rome, Via Firenze 1, 04019 Terracina, Italy; 3 Ophthalmology Department, Fondazione Policlinico Universitario A. Gemelli, IRCCS, 00168 Rome, Italy

**Keywords:** Nd laser, posterior capsulotomy, intraocular lens, hydrophobic acrylic, dysphotopsia, optical bench, point spread function, modulation transfer function, retinal light scattering, intraocular lens damage

## Abstract

Nd laser posterior capsulotomy is the standard treatment for posterior capsule opacification following cataract surgery. Although generally considered safe, inadvertent laser impacts on the intraocular lens (IOL) optic may induce permanent surface defects that contribute to postoperative dysphotopsias and reduced visual quality. This experimental study investigated the optical consequences of Nd laser-induced damage on two commercially available hydrophobic acrylic IOLs, focusing on retinal light distribution and optical image quality. Two hydrophobic acrylic monofocal IOL models, the CT LUCIA (Carl Zeiss Meditec) and the AcrySof IQ (Alcon), were mounted on a customized experimental holder and exposed to standardized Nd laser applications consisting of 5, 10, or 15 laser shots. Laser interactions were documented using the PhysioGo.Lite laser platform combined with infrared thermal imaging. Untreated IOLs served as controls. Optical performance was subsequently evaluated using a standardized optical bench according to ISO recommendations. Point spread function (PSF) and modulation transfer function (MTF) analyses were performed to quantify retinal image quality, light scattering, and optical degradation. Retinal light distribution was assessed using a high-resolution projection screen simulating the retinal image. Laser exposure produced permanent focal defects on the anterior optical surface of both IOL models, resulting in measurable optical degradation. Even the lowest laser exposure (five shots) generated detectable alterations in light propagation, characterized by increased peripheral light scattering, enlargement of the PSF halo, reduced central peak intensity, and irregular light distribution across the simulated retinal plane. Descriptively, increasing numbers of laser impacts were associated with more pronounced optical disturbances, particularly in the AcrySof IQ samples. MTF analysis demonstrated a reduction in optical performance across multiple spatial frequencies, indicating deterioration of image contrast and resolving power. Although both hydrophobic acrylic IOL models exhibited optical alterations after laser exposure, descriptive differences in the magnitude and distribution of light scatter suggested a possible influence of material composition, refractive index, and surface microarchitecture. These observations should be considered preliminary because of the limited sample size and absence of inferential statistical analysis. Under the present experimental conditions, Nd:YAG laser-induced pitting was associated with measurable structural and optical alterations in two hydrophobic acrylic IOL models. Surface defects alter retinal light distribution, increase forward light scatter, and reduce optical quality, providing a possible optical mechanism that may contribute to postoperative dysphotopsias, although clinical visual symptoms were not directly evaluated in this study. These findings highlight the importance of meticulous laser focusing on the posterior capsule to minimize inadvertent IOL damage and preserve postoperative visual quality. Further investigations combining optical bench analyses with patient-reported visual outcomes are warranted to better define the clinical significance of laser-induced IOL pitting.

## 1. Introduction

Cataract surgery is one of the most frequently performed surgical procedures worldwide and represents the standard treatment for lens opacification, the leading cause of reversible blindness globally [1]. Modern cataract surgery is routinely performed using phacoemulsification followed by implantation of an intraocular lens (IOL), providing excellent visual rehabilitation with high safety and efficacy [2,3]. Currently, four principal biomaterials are available for IOL manufacturing: polymethylmethacrylate (PMMA), hydrophilic acrylic, hydrophobic acrylic, and silicone [4,5].

One of the most common long-term complications following cataract surgery is posterior capsule opacification (PCO), commonly referred to as secondary cataract, which may progressively reduce visual acuity, contrast sensitivity, and overall visual quality, significantly affecting patients’ daily activities [6,7]. PCO develops because the complete removal of lens epithelial cells (LECs) during cataract extraction is impossible. Residual equatorial LECs retain proliferative potential, migrating toward the posterior capsule where they undergo proliferation, transdifferentiation into myofibroblasts, extracellular matrix deposition, and fibrotic remodeling, ultimately leading to visually significant capsule opacification [8,9,10,11,12,13].

The incidence of PCO is strongly influenced by patient age. Epidemiological studies report rates approaching 70% in patients younger than 40 years, approximately 37% in adults older than 40 years, and nearly 100% in pediatric patients, reflecting the greater proliferative capacity of younger lens epithelial cells [14,15].

Nd laser posterior capsulotomy remains the gold-standard treatment for visually significant PCO because it is rapid, minimally invasive, and highly effective in restoring visual function [16]. Despite continuous improvements in cataract surgical techniques and IOL design, approximately 15–33% of patients undergoing uncomplicated phacoemulsification eventually require laser capsulotomy during postoperative follow-up [17,18,19,20,21].

Although Nd capsulotomy is generally considered safe, one of its most frequent procedure-related complications is inadvertent laser impact on the implanted IOL optic rather than the posterior capsule, resulting in localized surface defects commonly referred to as IOL pitting [20]. Experimental investigations have demonstrated that the morphology and severity of laser-induced defects depend on the optical material. PMMA lenses typically develop characteristic “star-crater” lesions, whereas silicone IOLs exhibit a “splash-like” damage pattern following laser exposure [19,22].

The susceptibility of an IOL to laser-induced damage is influenced by several factors, including biomaterial composition, refractive index, polymer architecture, manufacturing process, and surface characteristics [23]. These variables determine the laser damage threshold, defined as the minimum laser energy capable of producing permanent structural alterations within the optic. Optical microscopy analyses have demonstrated that silicone IOLs exhibit the lowest resistance, with a damage threshold of approximately 0.37 mJ, followed by acrylic lenses (0.52–0.66 mJ), whereas PMMA lenses appear to be the most resistant, with reported thresholds around 0.68 mJ [24].

To date, no universally accepted laser energy threshold has been identified that reliably separates the energy required for effective capsulotomy from that capable of producing clinically relevant IOL damage [25,26,27,28,29]. Consequently, current recommendations emphasize the use of the lowest effective laser energy, accurate focusing posterior to the capsule, and meticulous aiming to minimize the risk of inadvertent IOL injury.

Fortunately, most cases of IOL pitting produce little or no clinically significant visual impairment. Nevertheless, when defects involve the visual axis or become extensive, patients may experience glare, halos, reduced contrast sensitivity, decreased visual acuity, or the perception of fixed opaque spots. In rare but severe cases, extensive optic damage may necessitate surgical IOL explantation and exchange [30,31].

While previous studies have primarily characterized the morphological appearance and physical properties of laser-induced IOL damage, considerably less is known about its functional optical consequences. In particular, the effects of laser pitting on retinal light distribution, forward light scatter, image quality, and optical performance remain incompletely understood. Previous experimental investigations have demonstrated that Nd:YAG laser impacts can produce material-dependent surface craters, microfractures, and irregularities in acrylic, silicone, and polymethyl methacrylate IOLs [29,32,33,34]. However, most available studies have focused primarily on damage morphology, laser-damage thresholds, or surface characterization. Objective optical consequences, including changes in point spread function, modulation transfer function, forward light scatter, contrast transmission, and retinal image quality, have been evaluated less extensively. Therefore, the relationship between the structural appearance of laser-induced pits and the resulting degradation of optical performance remains incompletely defined. In particular, although surface defects may theoretically increase straylight and alter contrast transmission, direct comparisons between untreated and laser-damaged IOLs using complementary structural and optical measurements remain limited. The present study was therefore designed as an exploratory optical bench investigation integrating SEM, thermographic imaging, digital beam-displacement analysis, PSF assessment, and MTF evaluation.

The CT LUCIA and AcrySof IQ models were selected because both are commonly used hydrophobic acrylic monofocal IOLs with the same nominal dioptric power, while differing in polymer formulation, refractive index, manufacturing characteristics, and surface microarchitecture. Their inclusion allowed an exploratory comparison between two clinically established hydrophobic acrylic platforms while limiting variability related to IOL category and refractive power.

Therefore, the purpose of the present experimental study was to investigate how Nd laser-induced pitting alters light transmission through hydrophobic acrylic intraocular lenses using a standardized optical bench model. Specifically, we evaluated whether laser-induced surface defects modify retinal light distribution and quantified the resulting optical degradation through point spread function (PSF) and modulation transfer function (MTF) analyses, providing objective evidence of optical alterations that may contribute to, but cannot by themselves demonstrate, the visual disturbances occasionally reported after Nd:YAG laser capsulotomy.

## 2. Materials and Methods

This experimental laboratory study was conducted through a collaboration between the Department of Ophthalmology and the Physics Laboratory of the “Gaetano Martino” University Hospital, Messina, Italy. The aim of the study was to investigate the structural and optical consequences of Nd:YAG laser-induced pitting in hydrophobic acrylic intraocular lenses (IOLs) using a multimodal experimental approach integrating scanning electron microscopy (SEM), infrared thermal imaging, optical bench analysis, and digital image processing.

The experimental setup included sixteen monofocal hydrophobic acrylic intraocular lenses, comprising eight CT LUCIA IOLs (Carl Zeiss Meditec, Jena, Germany) and eight AcrySof IQ IOLs (Alcon Laboratories Inc., Fort Worth, TX, USA), a customized IOL holder, a VISULAS YAG III laser system (Carl Zeiss Meditec, Jena, Germany) for the induction of laser pitting, and a PhysioGo.Lite laser platform (ASTAR Industry, Bielsko-Biała, Poland) for standardized optical illumination during thermographic acquisition, an optical bench equipped with a collimated LED light source, a retinal simulation screen consisting of a white A4 sheet (emissivity = 0.70), a complementary metal–oxide–semiconductor (CMOS) image sensor, and a FLIR T440bx infrared thermal camera (FLIR Systems, Wilsonville, OR, USA). Morphological evaluation of laser-induced defects was performed using scanning electron microscopy, whereas digital image processing and quantitative analyses were carried out using MATLAB R2024a (MathWorks, Natick, MA, USA).

### 2.1. Nd:YAG Laser Procedure

Laser-induced pitting was produced using a VISULAS YAG III Nd:YAG laser system (Carl Zeiss Meditec, Jena, Germany). Each IOL was positioned on a custom-designed holder and exposed to standardized laser applications using a pulse energy of 1.2 mJ, reproducing the energy levels commonly employed during posterior capsulotomy. All laser procedures were performed on isolated intraocular lenses mounted in air. No aqueous-filled model eye, balanced salt solution, or capsular bag simulation was used during laser exposure. For each IOL model, two lenses received five laser impacts, two received ten impacts, and two received fifteen impacts. Two additional untreated lenses of each model served as controls. All laser applications were performed under identical experimental conditions by the same experienced operator to ensure reproducibility and minimize procedural variability. The laser impacts were applied within the central optic at adjacent non-overlapping locations to reproduce multiple inadvertent laser hits while avoiding repeated impacts within the same crater.

### 2.2. Thermographic Image Acquisition

Following laser treatment, infrared images were acquired using a FLIR T440bx thermal imaging camera. During image acquisition, the PhysioGo.Lite laser platform (ASTAR Industry) was employed as a standardized optical illumination source. The system was operated under constant conditions with a spot size of 0.3 cm^2^, an output power of 200 mW, an irradiation time of 18 s, and an energy density of 12 J/cm^2^, thereby ensuring reproducible illumination for all experimental measurements. Thermal images were acquired at a frequency of 60 Hz over a 30 s acquisition period and stored in TIFF format before subsequent processing using MATLAB. Infrared thermography was not used to quantify heat-related material damage or temperature-dependent optical degradation. Its purpose was to provide a reproducible visualization of the spatial distribution of the transmitted light spot under standardized illumination conditions. The thermographic images were subsequently used for digital image registration, subtraction, and centroid-displacement analysis. Therefore, this component of the experiment assessed changes in light propagation rather than the thermal mechanism of Nd:YAG-induced IOL damage.

### 2.3. IOLs Characteristics

A total of sixteen monofocal hydrophobic acrylic intraocular lenses (IOLs) were included in the study, comprising eight CT LUCIA lenses (Carl Zeiss Meditec, Jena, Germany) and eight AcrySof IQ lenses (Alcon Laboratories Inc., Fort Worth, TX, USA). All IOLs had an identical refractive power of +19.0 diopters and were selected from the models routinely implanted at our institution. These models were selected because they represent two widely used hydrophobic acrylic monofocal IOL platforms with the same nominal refractive power but different proprietary polymer formulations, refractive indices, manufacturing processes, and surface characteristics. The comparison was intended to explore whether two clinically established hydrophobic acrylic platforms might exhibit different descriptive patterns of laser-induced structural and optical alteration. The study was not designed or powered to establish definitive superiority or material-specific resistance.

Each IOL was mounted on a custom-designed lens holder to ensure stable positioning and reproducible alignment throughout the experimental procedures. Laser treatment was subsequently performed according to the protocol described in Section 2.1. Untreated and laser-treated lenses were then subjected to morphological, thermographic, and optical bench analyses.

### 2.4. FLIR T440bx Thermal Imaging CAMERA

The FLIR T440 thermal imaging camera was utilized in this study. It is a long-wave thermal imager equipped with an uncooled microbolometer focal array. The spectral range of sensitivity spans from 7.5 to 13 μm, with a thermal sensitivity of 0.1 °C and an FPA (focal plane array) of 320 × 240 pixels. The thermal camera captured images at a rate of 60 Hz, with each acquisition lasting for 30 s. The images were initially acquired in .tiff format and subsequently converted to .jpeg to facilitate further post-processing.

### 2.5. Scanning Electron Microscope

The analysis to assess the morphological characteristics of the lenses under study was conducted using a scanning electron microscope, specifically the Jeol JMC-6000 (Jeol Co., Akishima, Tokyo, Japan). Each lens was air-dried prior to examination. The scanning was performed in high-vacuum mode, scanning each sample along the x-y directions. The electron beam was accelerated at 15 kV to achieve a magnification of 300×. After each acquisition, all scanned images were saved in Tagged Image File Format (.tiff) without any further processing.

### 2.6. Optical Bench, PSF, and MTF Measurements

Optical quality was evaluated using a customized optical bench comprising a collimated LED illumination source, an adjustable aperture, a custom-designed IOL holder, an image-acquisition plane, and a CMOS sensor. Each IOL was positioned with its optical axis aligned with the illumination and imaging axes. Untreated and laser-treated IOLs were evaluated under identical geometrical and illumination conditions. Optical measurements were likewise performed in air. Consequently, the refractive environment differed from that of an intraocular lens immersed in aqueous humor within the pseudophakic eye.

The optical bench configuration included a 3.0 mm pupil aperture and monochromatic illumination with a central wavelength of 546 nm. The distance between the IOL and the image-acquisition plane was set at 50 mm, while images were recorded using a CMOS sensor with a resolution of 1920 × 1200 pixels. All measurements were performed in a controlled dark environment to minimize background illumination, stray reflections, and variations in image contrast.

For each IOL, three repeated acquisitions were obtained. The lens was removed and realigned between consecutive acquisitions to account for potential positioning variability. Measurement repeatability was assessed by calculating the mean and standard deviation of the values obtained from the three acquisitions.

PSF images were acquired using a point-like illumination target positioned along the optical axis. The resulting images were analyzed in terms of central peak intensity, halo extension, radial light distribution, and the presence of peripheral light spikes. Where applicable, the full width at half maximum was calculated from the intensity profile passing through the PSF centroid.

MTF curves were derived from the acquired PSF images using Fourier-transform analysis and were evaluated over a spatial-frequency range of 0–100 cycles/mm. Numerical MTF values were extracted at 10, 20, 30, 50, and 100 cycles/mm. The untreated control lens for each model was used as the reference for calculating the relative percentage reduction in MTF after Nd:YAG laser exposure.

**Figure 1:**
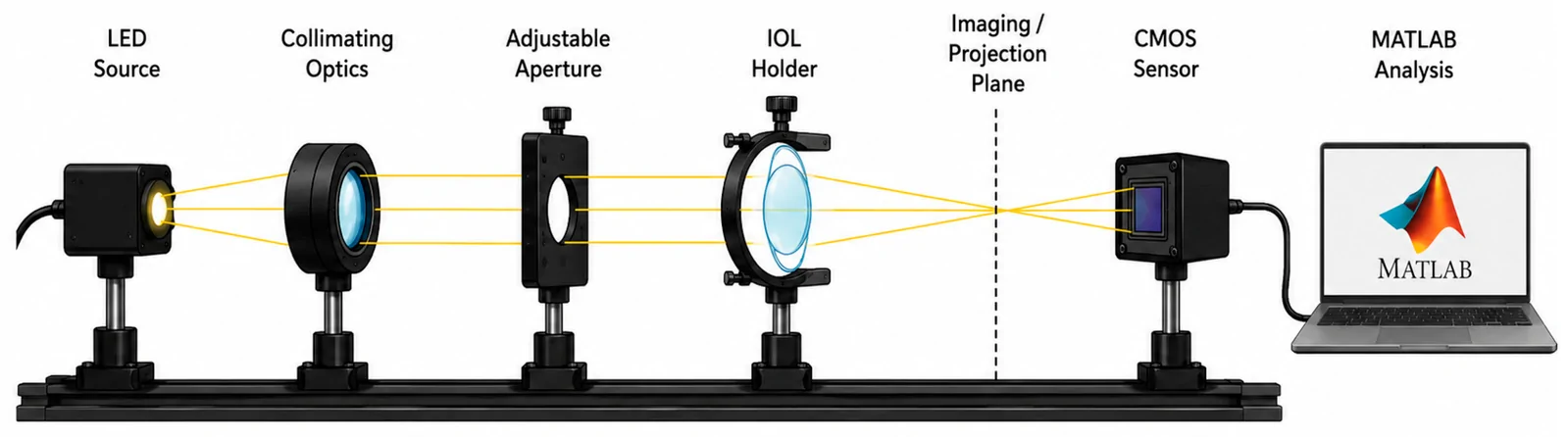
Schematic representation of the customized optical bench used for PSF and MTF assessment. The system included a collimated LED illumination source, an adjustable aperture, a custom-designed IOL holder, an imaging plane, and a CMOS sensor connected to MATLAB-based image-processing software.

The optical bench was configured according to the general principles described in ISO 11979-2:2024 [14] for laboratory evaluation of IOL optical properties. However, because the system was customized for this exploratory investigation and did not reproduce all elements of a certified model-eye testing platform, it should not be considered a fully ISO-compliant or certified optical bench. A schematic representation of the complete optical bench configuration is provided in Figure 1.

The available optical acquisition system was primarily designed for qualitative assessment of retinal light distribution and optical image degradation. Consequently, PSF analysis focused on descriptive features, including central peak intensity, halo enlargement, radial light distribution, and peripheral light spikes. Quantitative PSF metrics such as full width at half maximum (FWHM), Strehl ratio, and encircled energy were not calculated because the experimental setup was not specifically calibrated for validated quantitative PSF characterization.

### 2.7. MATLAB Software

MATLAB software was used for image registration, digital subtraction, and quantitative centroid analysis. All thermographic images were acquired at a resolution of 320 × 240 pixels. Before subtraction, images from untreated and laser-treated IOLs were converted to grayscale and normalized to the same intensity range.

To minimize errors caused by differences in camera or sample positioning, each image pair was subjected to rigid image registration. Registration was performed using fixed reference points within the acquisition field and translational correction along the x- and y-axes. No rotational or non-linear transformation was applied. Registered images were visually inspected before subtraction to confirm adequate alignment.

The image obtained from the corresponding untreated IOL was subtracted from the image obtained from each laser-treated IOL. Pixels with identical or closely similar intensity values were suppressed, whereas pixels showing intensity differences remained visible in the subtraction image.

The center of the transmitted light spot was identified using an intensity-weighted centroid algorithm. The centroid coordinates were calculated as

$$x_{c}=\frac{\sum xI\left(x,y\right)}{\sum I\left(x,y\right)}$$

and

$$y_{c}=\frac{\sum yI\left(x,y\right)}{\sum I\left(x,y\right)},$$

where $I\left(x,y\right)$ represents the pixel intensity at coordinates x and y.

The displacement between the untreated and laser-treated conditions was calculated as:

$$\Delta pixel=\sqrt{{\left(x_{treated}-x_{control}\right)}^{2}+{\left(y_{treated}-y_{control}\right)}^{2}}$$

This value represents the Euclidean displacement of the light-spot centroid on the image plane. Δ-pixel displacement was used as an experimental surrogate of altered light propagation and was not intended as a direct measure of retinal displacement, visual acuity, or clinical dysphotopsia.

To verify the stability of the centroid calculation, the analysis was repeated on 30 independent frames for each IOL condition. The reported Δ-pixel values represent the mean centroid displacement calculated across the 30 consecutive frames, while the corresponding standard deviation was used as an indicator of measurement repeatability. The same intensity threshold, image registration procedure, and centroid detection algorithm were applied to all images to ensure methodological consistency and minimize operator-dependent variability.

### 2.8. Statistical and Descriptive Analysis

This study was designed as an exploratory proof-of-concept laboratory investigation. Because only two IOLs were available for each model and experimental condition, no inferential statistical analysis was performed. Quantitative measurements are therefore reported descriptively as individual values or mean ± standard deviation, where replicate measurements were available. No *p*-values or claims of statistical significance were generated. Comparisons between IOL models and laser-shot conditions should consequently be interpreted as preliminary descriptive observations rather than statistically demonstrated differences.

## 3. Results

All results are presented descriptively because the number of IOLs per experimental condition was insufficient for inferential statistical analysis. The terms “increase,” “reduction,” and “difference” refer to observed numerical or qualitative patterns and should not be interpreted as statistically significant effects.

### 3.1. Scanning Electron Microscopy (SEM) Images

**Figure 2:**
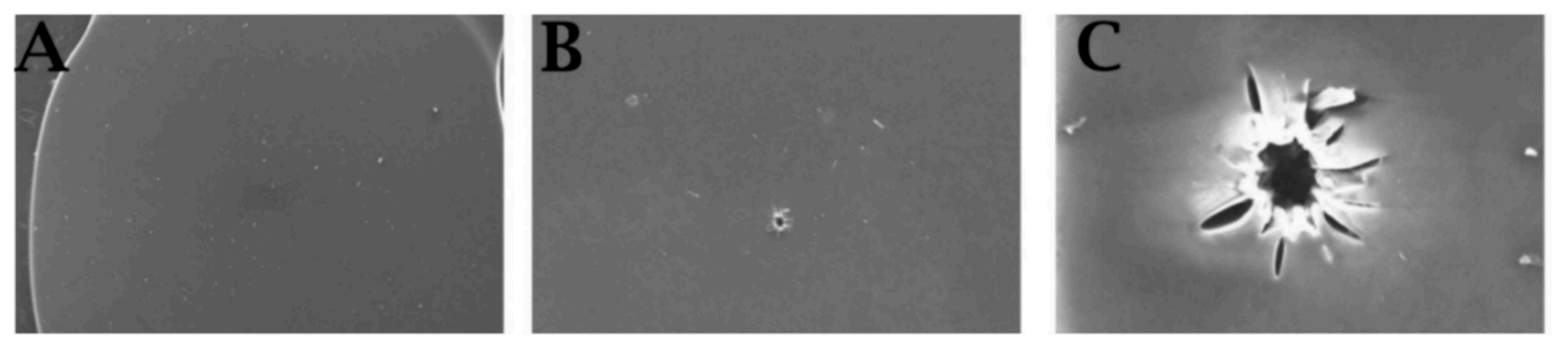
Representative scanning electron microscopy images of an untreated IOL (**A**), an IOL exposed to 5 Nd:YAG laser shots (**B**), and an IOL exposed to 15 Nd:YAG laser shots (**C**). The images qualitatively demonstrate localized surface disruption after laser exposure.

To highlight the damage to intraocular lenses (IOLs) post-Nd:Yag laser exposure, scanning electron microscopy (SEM) was utilized. This technique also allowed for the visual assessment of structural differences between Nd:Yag laser-impacted IOLs and unexposed (virgin) IOLs. Specifically, in Figure 2, the conditions of the virgin lens (A) can be compared to those of the lens exposed to the Nd:Yag laser 5 times (B) and 15 times (C).

In the virgin IOL, the shape is well-preserved, and the edges are regular. In the IOL exposed 5 times, the shape remains preserved, but a groove with irregular vesicles is visible. In the representative lens exposed to 15 laser shots, the damaged area appeared more extensive than that observed after 5 shots. Because the present SEM assessment was qualitative and based on a limited number of samples, this observation should be interpreted descriptively and not as a statistically demonstrated dose–response relationship.

### 3.2. Thermal Camera Record and MATLAB Processing

**Figure 3:**
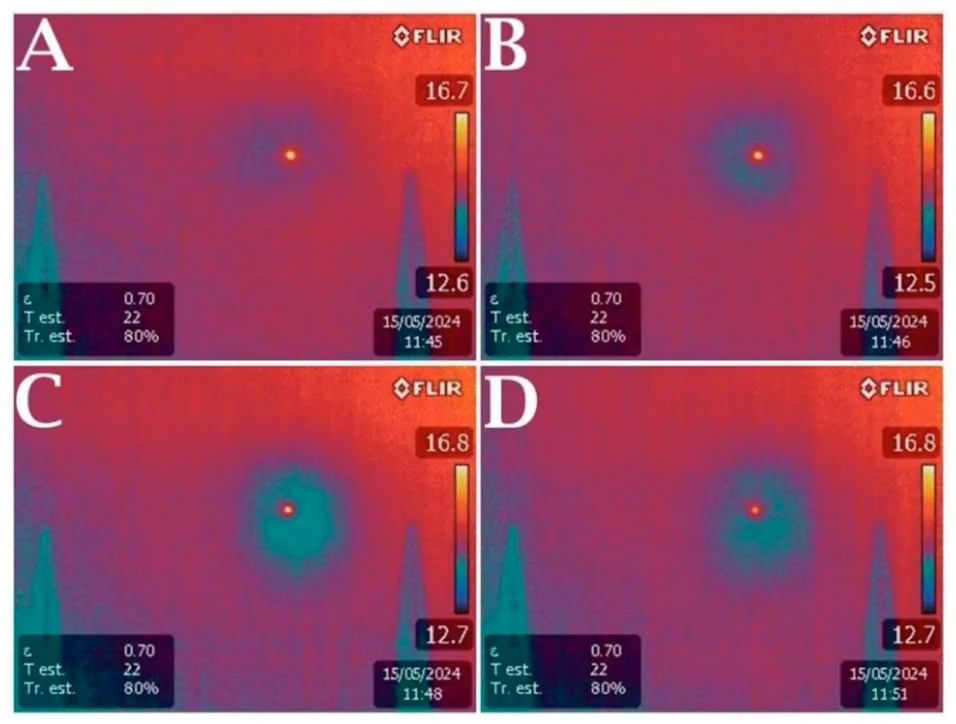
Representative thermographic images of CT LUCIA IOLs under standardized illumination: untreated control (**A**), 5 Nd:YAG laser shots (**B**), 10 shots (**C**), and 15 shots (**D**).

**Figure 4:**
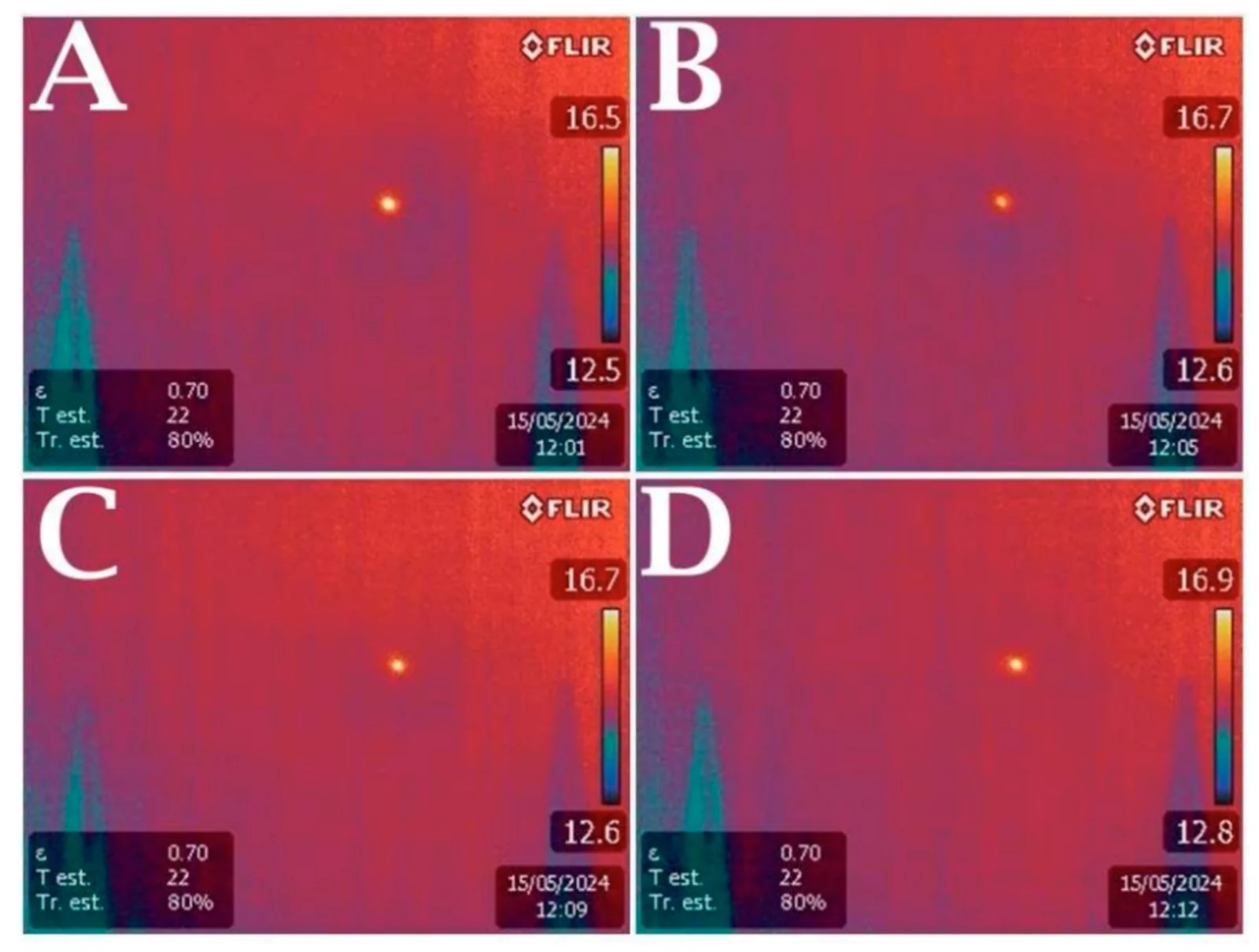
Representative thermographic images of AcrySof IQ IOLs under standardized illumination: untreated control (**A**), 5 Nd:YAG laser shots (**B**), 10 shots (**C**), and 15 shots (**D**).

The next step involved acquiring images using a thermal camera, which enabled the recording of infrared images in a digital format suitable for post-processing. In Figure 3A and Figure 4A, the position of the laser spot for the virgin lenses, corresponding to the Zeiss and Alcon IOLs, respectively, can be seen. The subsequent Figure 3B and Figure 4B pertain to infrared images of IOLs subjected to 5 Nd:Yag laser shots. Figure 3C and Figure 4C correspond to IOLs with 10 laser shots, while Figure 3D and Figure 4D show infrared images of IOLs exposed to 15 laser shots, associated with the Zeiss and Alcon IOLs, respectively.

**Figure 5:**
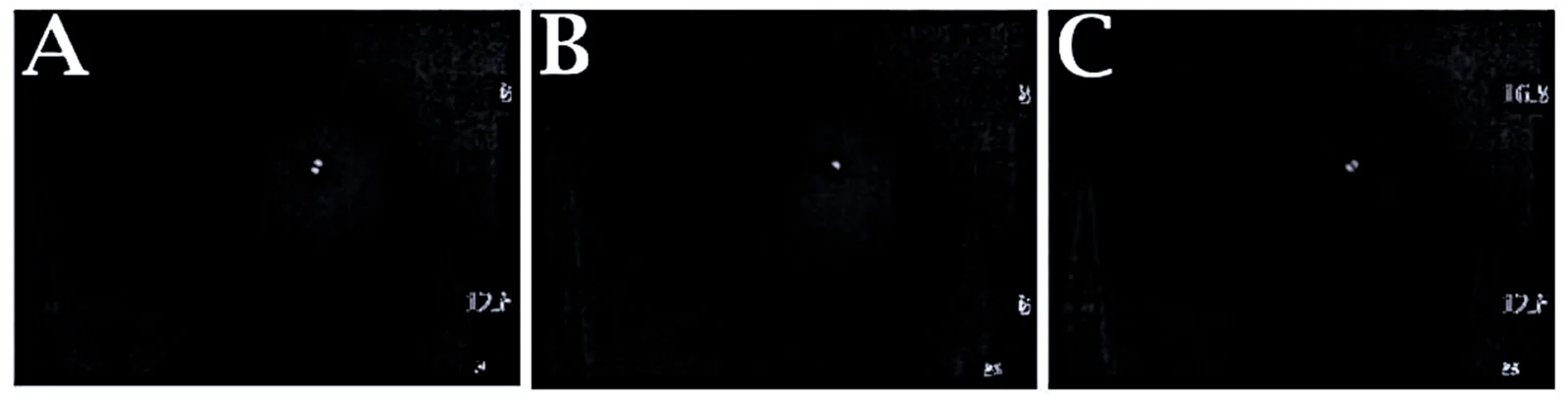
Registered digital subtraction images comparing untreated and laser-treated CT LUCIA IOLs: (**A**) after 5 Nd:YAG laser shots; (**B**) after 10 Nd:YAG laser shots; and (**C**) after 15 Nd:YAG laser shots. Bright regions indicate differences in transmitted light-spot position or intensity distribution.

During post-processing, digital image subtraction was performed using MATLAB software, as detailed in the “Materials and Methods” section. The digital subtractions of the images for Zeiss IOLs are shown in Figure 5. Figure 5A represents the difference between the virgin IOL and the IOL hit 5 times, Figure 5B shows the difference between the virgin IOL and the IOL hit 10 times, and Figure 5C illustrates the difference between the virgin IOL and the IOL hit 15 times.

**Figure 6:**
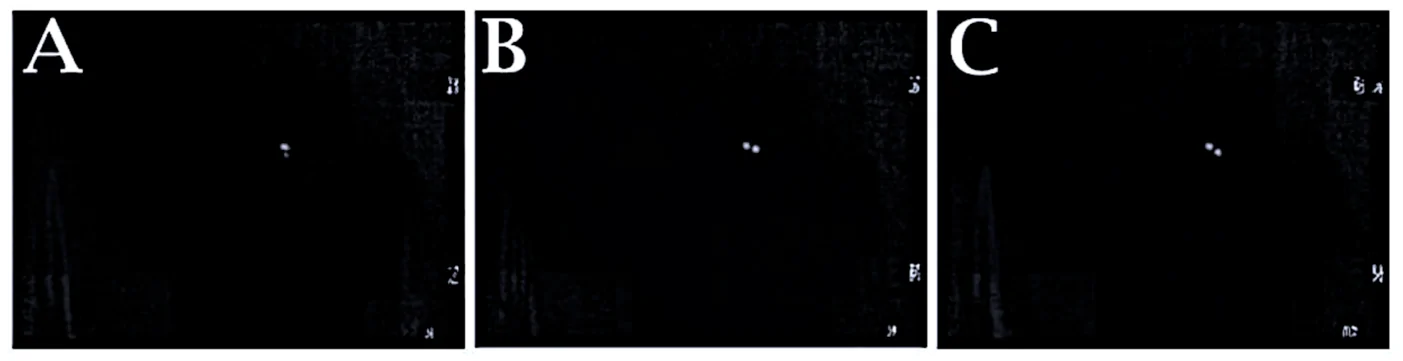
Registered digital subtraction images comparing untreated and laser-treated AcrySof IQ IOLs after 5, 10, and 15 Nd:YAG laser shots, from left to right (**A**–**C**). Bright regions indicate differences in transmitted light-spot position or intensity distribution.

Similar analyses were conducted for the Alcon IOLs, and the results of the digital thermographic image subtractions are presented in Figure 6. If there had been no displacement of the light spot, maintaining the same position in both the hit and virgin lenses, a black image would have resulted from the subtraction. However, in Figure 5 and Figure 6, the presence of two bright spots resulting from the subtraction of pixels with different intensities indicates an actual displacement of the light spot in the hit lenses compared to the virgin ones. Because the images were registered before subtraction, the paired bright regions in the subtraction maps were interpreted as differences in the position and intensity distribution of the transmitted light spot rather than simple camera or sample misalignment. Nevertheless, residual registration error cannot be completely excluded and represents a limitation of the analysis.

To evaluate the actual extent of the light spot displacement, the difference in pixels between the center of the spot on the virgin lens and the center of the spot on the impacted lens was calculated. Specifically, this difference is defined as Δ pixels.

**Figure 7:**
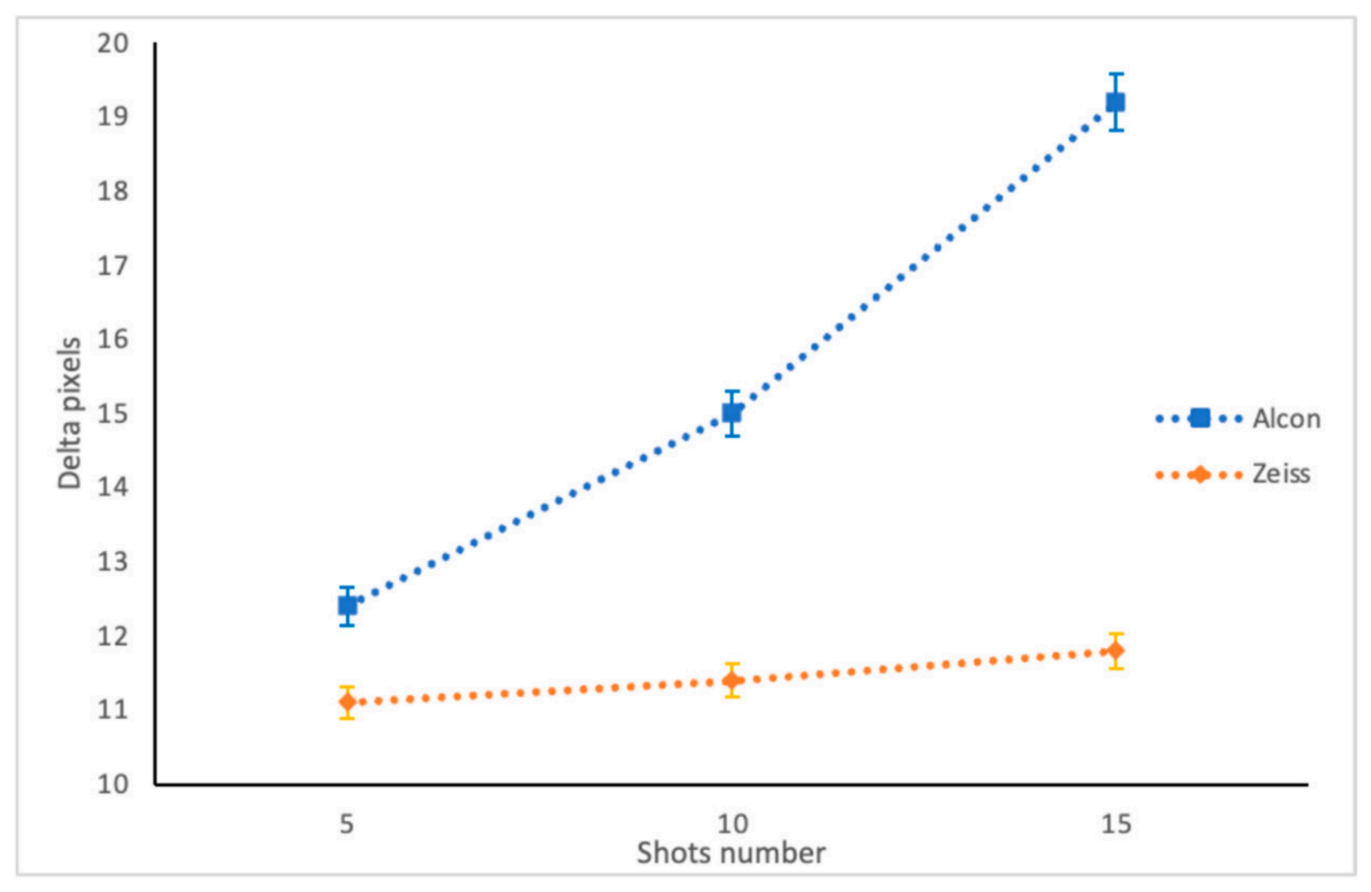
Descriptive variation in light-spot centroid displacement according to the number of Nd:YAG laser shots. Values are expressed as mean Δ-pixel displacement ± standard deviation for CT LUCIA and AcrySof IQ IOLs. No inferential statistical comparison was performed.

In Figure 7, the number of laser shots is plotted on the *x*-axis, and the Δ pixel values are plotted on the *y*-axis. This analysis was conducted for each of the two types of intraocular lenses, Zeiss and Alcon. The descriptive Δ-pixel values for CT LUCIA lenses were 13.2 ± 0.4 pixels after 5 shots, 13.6 ± 0.5 pixels after 10 shots, and 13.1 ± 0.3 pixels after 15 shots. The corresponding values for AcrySof IQ lenses were 11.8 ± 0.3, 14.5 ± 0.6, and 18.7 ± 0.7 pixels, respectively.

Observing the graph in Figure 7, it is noticeable that following the laser shots applied to the Zeiss lens (shown in red in Figure 7), there is a Δ pixel, indicating a displacement of the light spot. In the CT LUCIA samples, Δ-pixel values remained within a relatively narrow range across the three laser-exposure conditions. This descriptive pattern may suggest limited additional centroid displacement beyond the initial laser exposure, although no formal plateau could be statistically established.

Examining the trend for the Alcon IOLs (shown in black in Figure 7), similarly, there is a Δ pixel following the laser shots. Descriptively, the AcrySof IQ samples showed progressively higher Δ-pixel values with increasing numbers of laser shots. Given the limited number of lenses and experimental conditions, this pattern should not be interpreted as a statistically demonstrated linear relationship. The descriptive Δ-pixel values obtained for each IOL model and laser exposure are summarized in Table 1. Overall, CT LUCIA lenses exhibited relatively stable centroid displacement across the three laser-exposure conditions, whereas AcrySof IQ lenses showed progressively higher Δ-pixel values with increasing numbers of Nd:YAG laser shots. Because of the limited number of IOLs included in each experimental group, these observations should be interpreted as descriptive trends rather than statistically demonstrated differences (Table 1).

Δ-pixel displacement reflects a change in the position of the light-spot centroid on the experimental imaging plane. It does not directly correspond to a defined retinal distance, visual acuity change, or dysphotopsia severity. Therefore, it should be considered an experimental indicator of altered light propagation rather than a clinically validated outcome measure.

### 3.3. Optical Bench Analysis: PSF and MTF Evaluation

The optical performance of both hydrophobic acrylic intraocular lenses (IOLs), the CT LUCIA (Carl Zeiss Meditec) and the AcrySof IQ (Alcon Laboratories), was evaluated using Point Spread Function (PSF) and Modulation Transfer Function (MTF) analyses following Nd laser exposure. Measurements were performed after 5, 10, and 15 laser impacts and compared with untreated control IOLs. Control IOLs exhibited a compact and highly symmetric PSF characterized by a well-defined central intensity peak and minimal peripheral light scatter, consistent with optimal optical performance. Following Nd laser exposure, both IOL models demonstrated progressive deterioration of the PSF. Laser-induced surface defects produced enlargement of the central halo, increased forward light scatter, and the appearance of peripheral light spikes extending radially from the laser impact sites, indicating disruption of the normal retinal light distribution. PSF findings are presented as qualitative image-based observations because the experimental optical bench was not configured for validated quantitative PSF metrics such as FWHM, Strehl ratio, or encircled energy. Therefore, the present analysis focused on comparative changes in halo extension, central peak intensity, peripheral light scatter, and overall PSF morphology.

**Figure 8:**
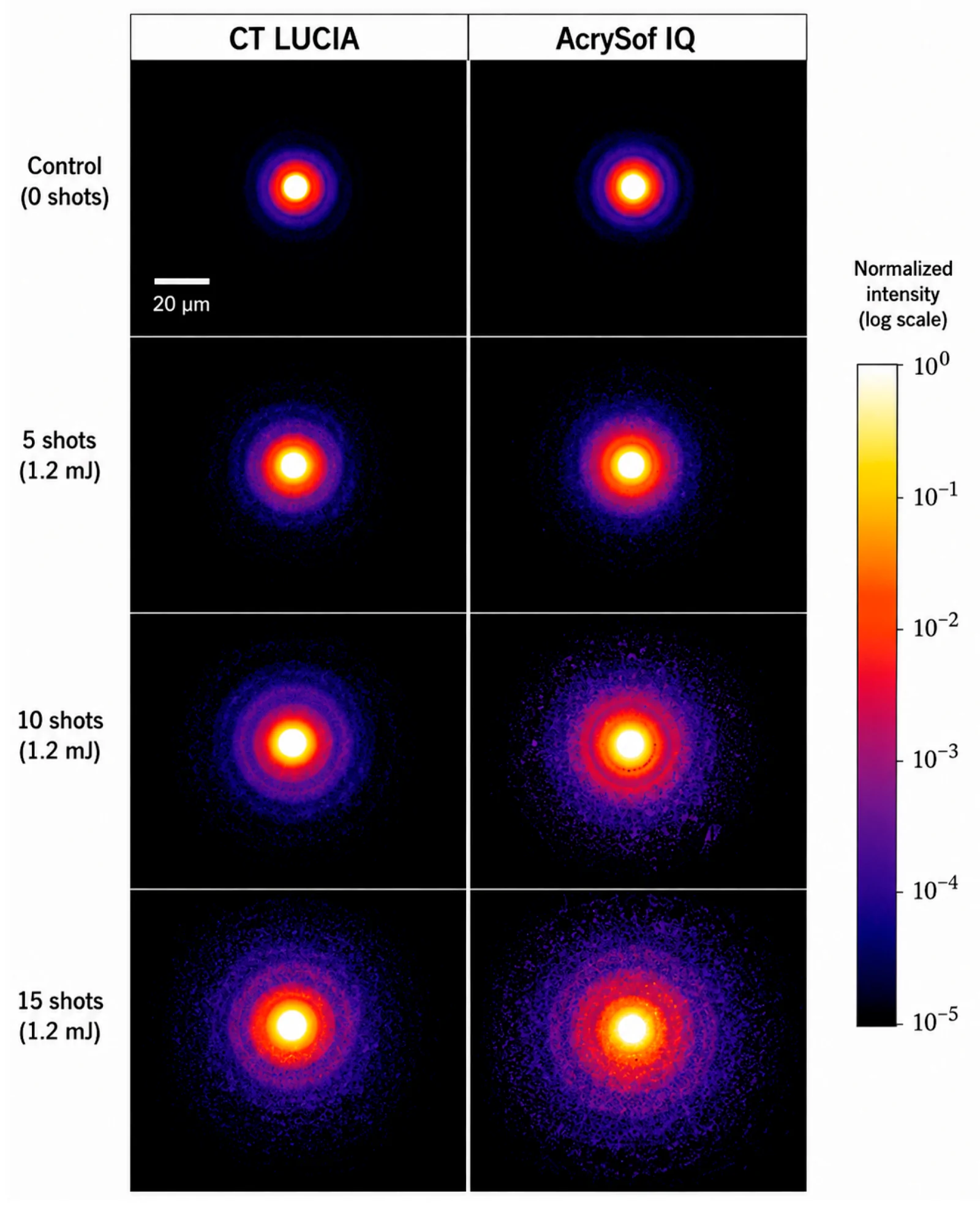
Representative point spread function (PSF) images obtained from the customized optical bench for untreated and Nd:YAG laser-treated hydrophobic acrylic intraocular lenses. CT LUCIA (left) and AcrySof IQ (right) lenses are shown under untreated conditions (0 shots) and after exposure to 5, 10, and 15 Nd:YAG laser shots (1.2 mJ). Images are displayed using a logarithmic intensity scale to enhance visualization of low-intensity scattered light. Progressive enlargement of the PSF halo and increased peripheral light scatter are observed with increasing laser exposure, particularly in the AcrySof IQ model. Because the experimental setup was not calibrated for quantitative PSF analysis, the images are presented for qualitative comparison only.

The two IOL models exhibited distinct patterns of optical degradation. In the CT LUCIA lenses, PSF enlargement was already detectable after five laser impacts and increased slightly after ten impacts. However, no substantial additional deterioration was observed after fifteen laser impacts, suggesting a plateau effect in optical degradation. In contrast, the AcrySof IQ lenses showed a progressive and nearly linear increase in PSF enlargement with each additional laser exposure. The extent of light diffusion increased proportionally with the number of laser impacts, in agreement with the progressive Δ-pixel displacement observed during digital image analysis, indicating cumulative optical impairment. Representative point spread function (PSF) images for untreated and Nd:YAG laser-treated CT LUCIA and AcrySof IQ intraocular lenses are shown in Figure 8. Compared with untreated controls, both IOL models demonstrated progressive enlargement of the PSF halo and increased peripheral light scatter with increasing numbers of laser shots. These changes were more evident in the AcrySof IQ samples after 10 and 15 laser impacts.

**Figure 9:**
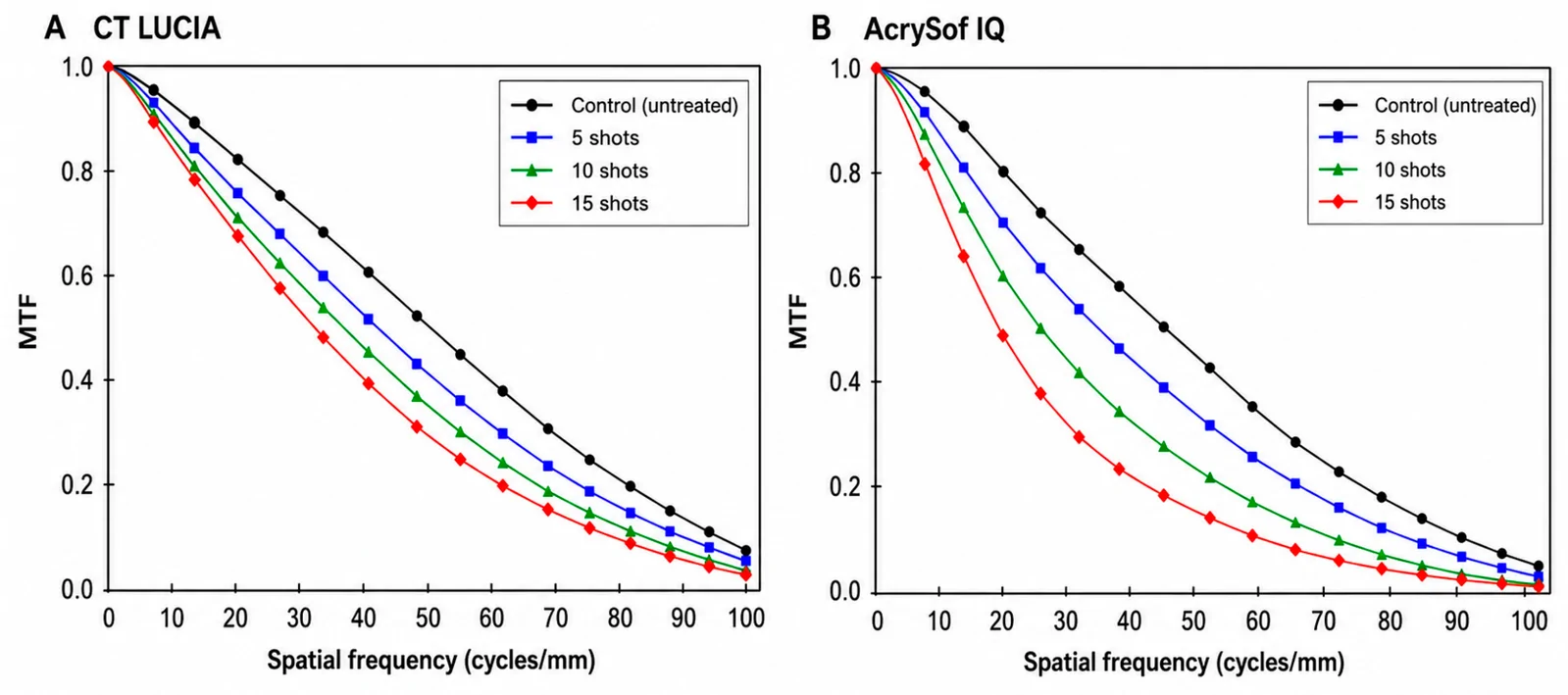
Complete modulation transfer function (MTF) curves for untreated and Nd:YAG laser-treated intraocular lenses: (**A**) CT LUCIA and (**B**) AcrySof IQ. Curves are shown across the full spatial-frequency range evaluated for untreated controls and after 5, 10, and 15 Nd:YAG laser shots. The data are presented descriptively; no inferential statistical comparison was performed.

Representative MTF curves obtained for untreated and Nd:YAG laser-treated CT LUCIA and AcrySof IQ IOLs are shown in Figure 9. Compared with untreated controls, all laser-treated IOLs demonstrated a progressive reduction in MTF across the evaluated spatial-frequency range, indicating deterioration of image contrast and optical performance. As illustrated in Figure 9A, CT LUCIA IOLs exhibited a modest reduction in MTF after five and ten laser impacts, with relatively limited additional deterioration following fifteen impacts. In contrast, the AcrySof IQ model (Figure 9B) showed a more pronounced progressive reduction in MTF with increasing laser exposure.

Compared with untreated controls, all laser-treated IOLs demonstrated decreased MTF values across the entire range of evaluated spatial frequencies, reflecting reduced image contrast and optical resolution. The magnitude of MTF degradation differed between the two hydrophobic acrylic materials. The CT LUCIA IOLs exhibited a modest reduction in MTF after five and ten laser impacts, followed by minimal additional deterioration after fifteen impacts, confirming the saturation phenomenon observed in the PSF analysis. Conversely, the AcrySof IQ lenses demonstrated a progressive decline in MTF that closely paralleled the increasing number of laser impacts. Representative MTF curves are shown in Figure 8. Because the optical bench was primarily designed to provide comparative qualitative assessment of optical degradation rather than complete quantitative optical characterization, numerical MTF values at individual spatial frequencies are not reported. Instead, the MTF curves provide a descriptive comparison of the progressive reduction in optical performance following Nd:YAG laser exposure. Visual inspection of the MTF curves indicates a progressive reduction in optical performance with increasing laser exposure in both IOL models. The apparent reduction was more pronounced in the AcrySof IQ samples than in the CT LUCIA samples under the present experimental conditions. However, because no quantitative MTF dataset was available and no inferential statistical analysis was performed, these observations should be interpreted descriptively.

Overall, the combined PSF and MTF analyses indicated measurable optical alterations after Nd:YAG laser-induced pitting under the present experimental conditions. Because no inferential statistical analysis was performed, these findings should be interpreted as descriptive rather than statistically significant.

## 4. Discussion

Cataract surgery with phacoemulsification and intraocular lens (IOL) implantation is currently one of the most frequently performed surgical procedures worldwide and remains the gold standard for the treatment of cataract [1]. Posterior capsule opacification (PCO) is the most common long-term postoperative complication and continues to represent the leading indication for Nd laser posterior capsulotomy [6,7,8]. Although Nd:YAG posterior capsulotomy is considered a safe and highly effective procedure for the treatment of posterior capsule opacification, postoperative visual complaints such as glare, halos, reduced contrast sensitivity, and persistent dysphotopsias continue to be reported in a subset of patients despite apparently uncomplicated procedures [6,7,8,20]. In many cases, the underlying mechanism responsible for these symptoms remains poorly understood, particularly when no clinically significant complications are evident on slit-lamp examination. Therefore, identifying the optical consequences of inadvertent IOL pitting may provide useful mechanistic information for understanding postoperative visual disturbances, although direct clinical relevance cannot be established without psychophysical testing and patient-reported outcomes [20,31]. A successful Nd capsulotomy depends on meticulous surgical technique, including accurate focusing on the posterior capsule, appropriate posterior offset (typically 100–250 μm), the use of the lowest effective laser energy, and minimization of the total number of laser pulses. Any anterior displacement of the focal point, excessive laser energy, or inaccurate aiming may result in unintended laser impacts on the IOL surface. The present findings indicate that even minimal laser misfocusing is sufficient to produce permanent structural damage capable of altering the optical performance of the implanted lens.

The primary objective of this study was to investigate whether laser-induced IOL pitting modifies the propagation of light through hydrophobic acrylic intraocular lenses and whether these alterations may influence retinal image formation. Using a multimodal experimental approach combining thermal imaging, scanning electron microscopy (SEM), optical bench analysis, and digital image processing, we demonstrated that laser-induced pits alter the normal optical pathway by deviating transmitted light from its expected trajectory. Thermal imaging combined with MATLAB-based digital subtraction analysis demonstrated measurable displacement of the transmitted light beam after Nd-laser exposure. Comparison between untreated and laser-treated IOLs consistently revealed displacement of the projected light spot, indicating that laser-induced surface irregularities modify the refractive behavior of the optic. Notably, this phenomenon was observed even after a limited number of laser impacts, suggesting that relatively small surface defects are sufficient to produce measurable optical alterations. From a clinical perspective, these findings suggest that even limited laser-induced surface defects may redirect incident light toward non-physiological retinal locations, thereby increasing forward light scatter without necessarily affecting conventional visual acuity. This mechanism may represent one possible contributor to persistent visual disturbances in patients with apparently minor IOL damage. However, the present ex vivo study did not evaluate visual function, contrast sensitivity, straylight perception, or patient-reported dysphotopsia and therefore cannot establish a causal relationship.

Descriptively, the two hydrophobic acrylic IOL models exhibited different optical response patterns after laser exposure. The AcrySof IQ lenses showed progressively greater displacement of the transmitted light beam (Δ-pixel analysis) with increasing numbers of laser impacts, whereas the CT LUCIA lenses demonstrated relatively stable spot displacement after the initial laser exposures. These preliminary observations raise the hypothesis that differences in polymer composition, refractive index, manufacturing process, or surface microarchitecture may influence the optical response to Nd:YAG-induced damage. However, the study was not designed to isolate the contribution of each material characteristic.

The optical bench analysis further supported these observations. PSF measurements demonstrated progressive enlargement of the central halo, increased forward light scatter, and the appearance of directional light spikes originating from laser-induced pits. Similarly, MTF analysis revealed a progressive reduction in optical quality following laser exposure, indicating deterioration of image contrast and spatial resolution. The greatest reduction in MTF was observed at intermediate spatial frequencies, which are particularly relevant for functional vision and contrast sensitivity in pseudophakic patients. In the limited samples evaluated, the AcrySof IQ lenses showed descriptively greater cumulative alterations in some optical outcomes than the CT LUCIA lenses. This observation should be considered preliminary and requires confirmation in larger experimental series.

Since both PSF enlargement and MTF reduction are closely associated with decreased retinal image quality and impaired contrast sensitivity, the present findings extend previous morphological observations by demonstrating their potential functional relevance. These optical changes provide a plausible experimental mechanism that could contribute to subjective complaints such as glare, halos, or reduced visual quality. Nevertheless, the study did not measure psychophysical visual performance or patient-reported symptoms, and no direct clinical correlation can therefore be inferred.

Previous studies of Nd:YAG-induced IOL damage have primarily focused on surface morphology and laser-damage thresholds. Newland et al. demonstrated material-dependent differences in damage formation among acrylic, PMMA, and silicone IOLs, while Borkenstein and Borkenstein characterized the morphology and optical-surface alterations produced by Nd:YAG impacts. Meduri et al. used SEM and X-ray spectrometry to document structural and compositional changes in PMMA IOLs after laser exposure. The present study extends these morphological observations by combining surface imaging with experimental assessment of light-spot displacement, PSF alteration, and MTF reduction. However, unlike standardized large-sample optical bench studies, the current investigation remains exploratory and does not establish statistically validated differences between materials.

Although most cases of IOL pitting are considered clinically insignificant, the present findings provide an optical explanation for the dysphotopsias occasionally reported after Nd capsulotomy, including glare, halos, reduced contrast sensitivity, and localized visual disturbances. Even when laser-induced pits do not directly involve the visual axis, the resulting increase in forward light scatter may contribute to subtle degradation of retinal image quality that is not readily detectable using conventional visual acuity testing alone.

Patient satisfaction after cataract surgery increasingly depends on visual quality rather than visual acuity alone. Modern pseudophakic patients frequently report subtle visual disturbances despite excellent Snellen acuity. Therefore, preservation of optical quality has become a major objective of contemporary cataract surgery and postoperative management. The present findings suggest that preventing even limited Nd:YAG-induced IOL damage may contribute to maintaining postoperative optical quality and reducing the risk of dysphotopsias.

Several limitations of this study should be acknowledged. First, the experimental model employed a simplified optical bench incorporating a retinal simulation screen and thermographic imaging, which cannot fully reproduce the complexity of retinal image formation or the optical and perceptual environment of the pseudophakic human eye. In particular, the experimental setup did not account for physiological variables such as tear-film instability, corneal and internal aberrations, aqueous and vitreous light propagation, pupil size and dynamics, IOL decentration or tilt, retinal sampling, neural processing, neuroadaptation, or the subjective perception of glare, halos, and other dysphotopsias. Consequently, the imaging plane used in this study should not be regarded as a direct surrogate for the human retina. Nevertheless, the highly controlled laboratory configuration enabled reproducible comparisons of the relative optical alterations induced by Nd:YAG laser damage. A major limitation of this study is that both the Nd:YAG laser treatment and the optical measurements were performed in air rather than in a fluid-filled model eye. Consequently, laser-induced pit morphology, cavitation dynamics, and optical scattering may differ from those occurring in vivo. Therefore, the present findings should not be directly extrapolated to the pseudophakic human eye.

Second, only two IOLs were available for each model and laser-exposure condition, precluding inferential statistical analysis. Accordingly, all findings should be interpreted as descriptive observations, and the apparent differences between CT LUCIA and AcrySof IQ lenses should be considered preliminary and hypothesis-generating rather than definitive evidence of material-dependent susceptibility.

Third, the study was conducted under ex vivo laboratory conditions, and Δ-pixel displacement represents an experimental image-plane parameter that has not been validated against clinically relevant functional outcomes such as retinal image displacement, contrast sensitivity, straylight, or patient-reported dysphotopsias. Therefore, direct extrapolation of these findings to postoperative visual performance should be avoided.

Another limitation is that the experimental setup was not calibrated to provide validated quantitative PSF metrics, such as Strehl ratio, full width at half maximum (FWHM), or encircled energy. Consequently, PSF analysis was restricted to qualitative assessment of changes in halo extension, central peak intensity, and overall light distribution. Similarly, complete numerical MTF datasets at individual spatial frequencies were not available. Optical performance was therefore evaluated descriptively using representative MTF curves rather than comprehensive quantitative optical characterization. Future investigations should employ standardized optical bench systems capable of generating validated quantitative PSF and MTF measurements.

Finally, only two hydrophobic acrylic monofocal IOL models were investigated, limiting the generalizability of the present findings to other IOL materials and designs. Despite these limitations, this study combines scanning electron microscopy, thermographic imaging, MATLAB-based image analysis, PSF assessment, and MTF evaluation within a single experimental platform, providing a comprehensive qualitative characterization of the structural and optical consequences of Nd:YAG laser-induced IOL pitting and generating hypotheses for future quantitative and clinical investigations.

To our knowledge, it is among the first investigations to combine scanning electron microscopy, infrared thermal imaging, digital image subtraction, PSF, and MTF analyses within a single experimental model to comprehensively evaluate the structural and functional consequences of Nd laser-induced IOL damage. This statement refers specifically to the combined use of these modalities within a single exploratory experimental protocol and does not imply that PSF, MTF, or optical effects of IOL damage have never been investigated previously.

Unlike previous studies that primarily described the morphology of laser-induced pits, the present work provides descriptive experimental evidence that laser-induced surface alterations are accompanied by measurable changes in light distribution and optical bench performance. Furthermore, the quantitative assessment of light beam displacement using Δ-pixel analysis provides an objective parameter linking physical surface damage with altered optical behavior. The comparative evaluation of two widely used hydrophobic acrylic IOLs also suggests material-dependent susceptibility to laser-induced optical degradation, which may have implications for future IOL design and material engineering.

## 5. Conclusions

In this exploratory optical bench study, Nd:YAG laser-induced pitting produced measurable structural and optical alterations in two hydrophobic acrylic intraocular lens (IOL) models, affecting transmitted light distribution, Δ-pixel displacement, PSF appearance, and MTF performance. Because of the limited sample size and the absence of inferential statistical analysis, these findings should be regarded as descriptive and hypothesis-generating. Although different patterns of optical alteration were observed between the two IOL models, the present data do not support definitive conclusions regarding material-dependent susceptibility.

Furthermore, as both laser exposure and optical assessment were performed under air-based laboratory conditions, the results cannot be directly extrapolated to the pseudophakic human eye. Nevertheless, these findings emphasize the importance of meticulous laser focusing, appropriate posterior offset, and the use of the lowest effective laser energy during Nd:YAG capsulotomy to minimize inadvertent IOL damage. Further studies using standardized model-eye systems and clinical investigations are warranted to clarify the clinical relevance of these experimental observations.

## Figures and Tables

**Table 1:** Descriptive Δ-pixel displacement according to the IOL model and the number of Nd:YAG laser shots.

IOL Model	5 Shots	10 Shots	15 Shots
CT LUCIA	13.2 ± 0.4	13.6 ± 0.5	13.1 ± 0.3
AcrySof IQ	11.8 ± 0.3	14.5 ± 0.6	18.7 ± 0.7

Values are presented as mean ± standard deviation of repeated image acquisitions. No inferential statistical comparison was performed because of the limited number of IOLs per condition.

## Data Availability

The datasets generated and analyzed during the current study are not publicly available due to institutional privacy regulations but are available from the corresponding author on reasonable request.

## Abbreviations

BCVA	Best-Corrected Visual Acuity
CMOS	Complementary Metal–Oxide–Semiconductor
FA	Forward Light Scatter
v	Intraocular Lens
IR	Infrared
ISO	International Organization for Standardization
LED	Light-Emitting Diode
LEC	Lens Epithelial Cell
MATLAB	Matrix Laboratory
MTF	Modulation Transfer Function
Nd:YAG	Neodymium-Doped Yttrium Aluminum Garnet
PCO	Posterior Capsule Opacification
PMMA	Polymethyl Methacrylate
PSF	Point Spread Function
SEM	Scanning Electron Microscopy
